# The Patient Acceptable Symptom State for Commonly Used Patient-Reported Outcomes After Nonoperative Management of Hip Femoroacetabular Impingement Syndrome

**DOI:** 10.1177/03635465261421191

**Published:** 2026-02-24

**Authors:** Graeme Hoit, Daniel B. Whelan, Valerie Lemieux, Brent Bates, Tim Dwyer, John Theodoropoulos, Jaskarndip Chahal

**Affiliations:** † Division of Orthopaedic Surgery, Department of Surgery, University of Toronto, Toronto, Ontario, Canada; ‡ Institute of Health Policy, Management and Evaluation, Dalla Lana School of Public Health, University of Toronto, Toronto, Ontario, Canada; § Division of Orthopaedic Surgery, Women’s College Hospital, Toronto, Ontario, Canada; ‖ University of Toronto Orthopaedic Sports Medicine, University of Toronto, Toronto, Ontario, Canada; Investigation performed at the University of Toronto, Toronto, Ontario, Canada

**Keywords:** Patient Acceptable Symptom State, patient-reported outcome measures, psychometric properties, femoroacetabular impingement syndrome, hip, nonoperative

## Abstract

**Background:**

Patient Acceptable Symptom State (PASS) values for commonly used patient-reported outcome measures are known for operatively treated patients with femoroacetabular impingement syndrome (FAIS) but have not been established for those undergoing nonoperative treatment.

**Purpose:**

First, to determine the PASS for International Hip Outcome Tool–33 (iHOT-33), Hip Outcome Score–Activities of Daily Living (HOS-ADL), and pain visual analog scale (VAS) in patients with FAIS treated nonoperatively; second, to assess the baseline factors that were associated with achieving PASS.

**Study Design:**

Cohort study (diagnosis); Level of evidence, 2.

**Methods:**

Patients with FAIS who were treated nonoperatively with an expert-validated physical therapy protocol at 2 academic centers were prospectively enrolled. Patients completed the iHOT-33, HOS-ADL, and pain VAS at baseline and 6 months after beginning treatment. Receiver operating characteristic curve analyses were conducted to determine PASS cutoff points. Multivariable regression analyses were performed to determine the association of patient factors, radiographic markers, and disease severity on the likelihood of achieving PASS.

**Results:**

Of the 214 patients enrolled, 121 (57%) were female, and the mean age was 34 years. The median symptom duration before beginning the prescribed physical therapy regimen was 24 months. The mean (SD) iHOT-33 score was 42.9 (16.7) at baseline and 54.2 (22.0) at 6 months. The iHOT-33 score for PASS is 50 (specificity, 91%; sensitivity, 82%; area under the curve [AUC], 0.95). The mean (SD) HOS-ADL was 72.9 (16.0) at baseline and 77.1 (17.1) at 6 months. The HOS-ADL score for PASS is 66 (specificity, 81%; sensitivity, 86%; AUC, 0.90). The mean (SD) pain VAS score was 53.7 (23.1) at baseline and 41.9 (SD25.4) at 6 months. The pain VAS score for PASS is 36 (specificity, 67%; sensitivity, 85%; AUC, 0.87). Patient age was significantly predictive of achieving PASS for iHOT-33 (odds ratio, 1.04; 95% CI, 1.01-1.08). Patients with higher baseline iHOT-33 scores were more likely to achieve PASS for all patient-reported outcome measures (*P* < .005). Otherwise, patient factors, radiographic markers, and symptom duration were not associated with achieving PASS.

**Conclusion:**

This study determined the PASS values for iHOT-33 (PASS = 50), HOS-ADL (PASS = 66), and pain VAS (PASS = 36) for patients with FAIS treated nonoperatively with an exercise-based, core-focused physical therapy program. These values can be utilized by clinicians in counseling individual patients to anticipated outcomes and by investigators for future nonoperative-focused outcomes research.

Femoroacetabular impingement syndrome (FAIS) is a condition caused by abnormal bone structure of the hip joint causing impaired clearance between the femoral head-neck junction and the acetabulum, leading to a painful hip condition linked to the development of hip osteoarthritis.^1,9,12^ The overall prevalence of FAIS has been estimated to be 10% to 15% of the population, most of whom are young active individuals aged 20 to 45 years.^37^ Treatments include nonoperative physical therapy–based approaches and surgical treatments, primarily through arthroscopic osteochondroplasty, often with labral and chondral surgery. A modern treatment algorithm for FAIS and optimal surgical patient selection remains contested, although most experts agree that a nonoperative regimen should be the initial treatment offered.^13^

Patient-reported outcome measures (PROMs) are often used to compare treatment outcomes in patients with FAIS. For the young active population with nonarthritic hip disorders, commonly used PROMs include the International Hip Outcome Tool–33 (iHOT-33), the Hip Outcome Score (HOS), and pain visual analog scale (VAS), among others. However, these PROMs are all expressed as continuous data at the group level, which can be difficult to interpret and challenging to translate to the responses of individual patients.^31,34^

The Patient Acceptable Symptom State (PASS) is a useful psychometric estimate that defines a numeric value—a specific score or threshold rather than a degree of change—above and below which patients consider themselves well and unwell, respectively.^17,33^ This allows for the interpretation of PROMs in the context of the individual patients’ perception of their disease state and allows for comparison of treatment arms in clinical research. PASS thresholds have been defined for surgical treatment of FAIS^16,18,35^; however, the PASS thresholds may meaningfully differ among different treatment regimens. Patients’ perception of their personal level of investment and associated risk in a treatment and, consequently, their expectations may affect the symptomatic state in which they consider themselves to be well.^30^ Patients who undergo operative management often perceive that they have been subjected to greater risks, pain, and life disturbances as compared with those who have been managed without surgery. Furthermore, the PASS for pain VAS has been determined for chronic conditions including hip and knee osteoarthritis and ankylosing spondylitis^31-34^ but not for young active patients with FAIS. Accordingly, it is important to define the threshold of acceptability of treatment outcomes for nonoperatively treated patients with FAIS.

The objective of this study was to determine the PASS of commonly used PROMs in patients undergoing nonoperative management of FAIS. Determining the PASS for these instruments would provide a useful clinical endpoint for decision-making at the individual level and outcome assessment in future studies. We hypothesized that reliable estimates of PASS values could be established using an anchor-based approach for the aforementioned treatment population. The secondary aim of the study was to determine the association between baseline factors and achieving PASS in each measure.

## Methods

### Study Design

This is an analysis from a prospective longitudinal cohort study conducted at 2 academic care centers. Institutional review board approval was obtained before the start of the study at both participating centers (WCH: 2018-0154-E; SMH: 20-013).

### Participants

Adult patients (age, 16-55 years) were enrolled if they had a diagnosis of FAIS and labral tear on magnetic resonance imaging and were treated nonoperatively with HipFit10, an expert-validated physical therapy protocol. In accordance with the Warwick consensus, diagnosis of FAIS was based on a triad of symptoms, physical examination, and imaging findings.^12^ Patients were excluded if they had confounding diagnoses (alternative hip diagnoses, spine pathology, and pain disorders), previous hip surgery, an inability to participate in exercise therapy, or potential secondary gain (workplace insurance claim, motor vehicle collision claim, or other medicolegal claim related to their hip pain).^12,21,25^ Complete inclusion and exclusion criteria are listed in Appendix I, available in the online version of this article.

### Procedures

All hip referrals to 1 of 4 participating fellowship-trained hip arthroscopic surgeons from August 2019 to October 2021 were screened for eligibility. Patients meeting inclusion criteria were approached by a research assistant for participation. Those who wished to enroll completed a demographic form (age, sex, body mass index [BMI], employment status, smoking status, baseline activity level [modified Tegner Activity Scale^3^]) and 3 baseline PROM questionnaires: HOS–Activities of Daily Living (HOS-ADL), iHOT-33, and pain VAS.

### Treatment

All participants were enrolled in HipFit10: a 6-month exercise-based, core-focused home exercise program with physical therapist involvement. The HipFit10 program is an expert-validated exercise program designed to treat patients with FAIS. Its creation was based on best evidence for initial treatment of FAIS, and it incorporated exercises from previously reported exercise protocols that demonstrated effectiveness.^2,12^ The rationale was to compile a practical, time-efficient, and easily understandable home strengthening program that was extension based to avoid impingement moments and that focused on strengthening the muscles that preferentially position the pelvis in posterior tilt—the abdominal muscles, glutes, and hamstrings.^13,26^ The program consists of 10 exercises of which participants are instructed to perform as many repetitions as possible in a 45-second period, resting as necessary, before transitioning to the next exercise. This format allows for an increasing number of repetitions as patients become stronger. Beginner modifications and extra challenges are provided for patients to modify the exercises to suit their levels of fitness. Patients were encouraged to perform the exercises at least 4 times per week. Each patient met with a physical therapist at the time of enrollment where the exercises were demonstrated and modifications were made on an individual basis as required. Participants were provided a handout of the instructions and exercises, as well as the link to the HipFit10 website (www.hipfit10.com). A second visit with a physical therapist was scheduled at the 4-week mark to revisit the exercises and make corrections or modifications as required.

### Patient-Reported Outcome Measures

The outcome measures evaluated in this study include the iHOT-33, the HOS-ADL, and the pain VAS. The iHOT-33 is a patient-derived measure of hip-related functional disability and quality of life. It contains 33 items pertaining to patients’ symptoms, functional impairments, sporting activities, job-related concerns, and social and emotional well-being, and it was developed to focus on items deemed important to a young active individual. Patients are asked to rate their level of impairment or pain as it pertains to their hip with specified activities on a scale. The maximum score is 100, with the higher score indicating less impairment.^20^ The HOS-ADL assesses the effect of patients’ hip disability on activities of daily living through 17 questions with a maximum score of 100, with a higher score indicating less impairment.^15^ The pain VAS is commonly used to evaluate pain levels in a variety of orthopaedic conditions. The pain VAS question utilized for the purpose of this study was “Below is scale on which no pain is marked as 0 and unbearable pain is marked as 100. Indicate on this scale the amount of pain in your affected hip.”^31-34^ These PROMs were administered at baseline and at 6 months after initiating the HipFit10 program.

### Standardized Measures

An anchor-based approach was used to determine the PASS. At 6 months after starting the physical therapy program, patients were asked the following binomial (yes vs no) question: “Taking into account all the activities you have during your daily life, your level of hip pain, and also your functional impairment, do you consider that your current state is satisfactory?”^33^

### Statistical Analysis

A receiver operating characteristic (ROC) curve analysis was utilized, and the cutoff point that maximized the Youden index defined the PASS threshold score for each PROM among patients who considered their state satisfactory.^11^ A post hoc analysis assessed the reliability using the area under the curve (AUC). An AUC value of 0.7 to 0.8 was regarded as satisfactory per the anchor question, and an AUC value of 0.8 to 0.9 was regarded as excellent.^6^ Multivariable logistic regression models were utilized to determine the independent association of baseline variables and the achievement of PASS for each of the 3 PROMs. We included variables that have been shown to be associated with outcomes in patients with FAIS, including patient factors (age,^8,14^ sex,^23^ BMI,^5^ and modified Tegner Activity Scale^24^), radiographic markers (impingement type,^27^ Tönnis classification grade,^4^ alpha angle,^29^ and lateral center-edge angle^36^), and disease severity (symptom duration^8^ and presenting symptom severity [baseline iHOT-33 score]^19,22^). The alpha level was set to .05, and *P* values less than or equal to the predetermined critical threshold (.05) were considered statistically significant. All statistical analyses were performed in SAS Version 9.3 (SAS Institute Inc).

## Results

**Figure 1.**
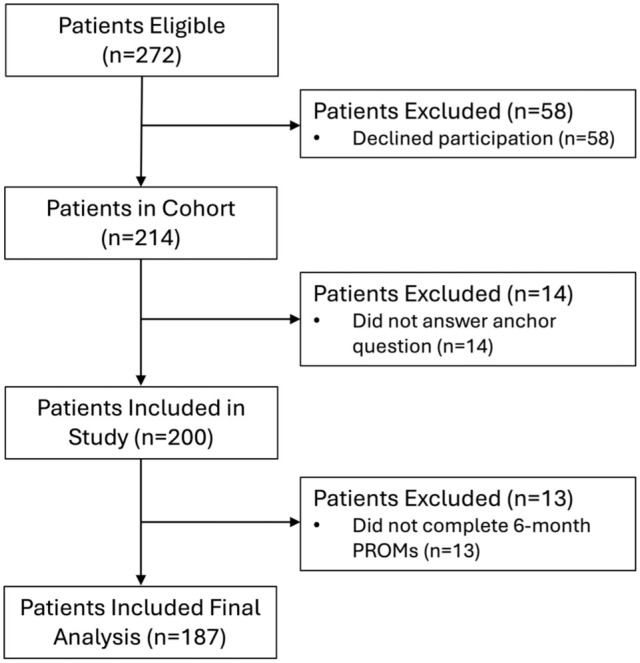
Patient flow diagram. PROM, patient-reported outcome measure.

In total, 214 patients agreed to participate in the study: 121 were female (57%); the mean (SD) age and BMI were 34 (10) years and 25.5 (4.9) kg/m^2^. The median symptom duration before enrollment was 24 months (range, 5-240). The mean baseline modified Tegner Activity Score was 5.3 (SD, 1.8; range, 1-9). There were no missing baseline data (Table 1). Of the 214 patients, 200 answered the anchor question (93%), and 187 completed their 6-month PROMs (87%) and were included in our final analysis (Figure 1).

**Table 1:** Baseline Characteristics of Study Population^*a*^

	All Patients (N = 200)	Anchor “Yes” (n = 98)	Anchor “No” (n = 102)
Patient characteristic			
Age, y	34.6 ± 10.2	34.6 ± 10.2	34.7 ± 10.2
Sex: female	121 (57)	58 (59)	55 (53)
BMI, kg/m^2^	25.5 ± 4.9	24.4 ± 3.8	26.2 ± 5.4
Modified Tegner Activity Scale	5.3 ± 1.8	5.9 ± 1.7	4.8 ± 1.8
Radiographic markers			
Impingement type			
Cam	79 (40)	43 (44)	36 (35)
Pincer	53 (27)	25 (26)	28 (27)
Mixed	68 (34)	30 (31)	38 (37)
Tönnis arthritis grade			
Grade 0	136 (68)	67 (68)	69 (68)
Grade 1	64 (32)	31 (32)	33 (32)
Alpha angle, deg	61.5 ± 12.4	61.9 ± 12.8	61.3 ± 12.2
Lateral-center edge angle, deg	35.4 ± 7.1	34.7 ± 7.3	36.2 ± 7.1
Disease severity			
Symptom duration, mo	43.5 ± 44.8	39.3 ± 45.1	49.4 ± 46.1
Baseline iHOT-33	42.9 ± 16.7	50.0 ± 15.7	35.1 ± 1.8
Lowest tertile	68 (34)	16 (24)	52 (51)
Middle tertile	68 (34)	33 (34)	35 (34)
Highest tertile	64 (34)	49 (50)	15 (15)

a Data presented as Mean ± SD or No. (%). BMI, body mass index; iHOT-33, International Hip Outcome Tool–33.

All PROM scores improved from baseline to 6 months with exercise-based therapy (Table 2). Of the 200 patients who responded to the anchor question, 98 (49%) responded yes and 102 (51%) no. The mean PROM scores of patients who answered yes versus no to the anchor question can be found in Table 3. Based on the ROC curve analysis, the PASS estimate for iHOT-33 was 50 points; for HOS-ADL, 66 points; and for pain VAS, 36 points. After 6 months of exercise therapy, 55% of patients achieved PASS for iHOT-33, and 49% achieved PASS for HOS-ADL and pain VAS. The percentage of patients presenting with baseline scores above PASS was highest for HOS-ADL (38%) and lowest for pain VAS (26%).

**Table 2:** Patient-Reported Outcome Measure Scores at Baseline and 6 Months^*a*^

Outcome Measure	Baseline			6 mo		
	Mean (SD)	Median (IQR)	Met PASS, %	Mean (SD)	Median (IQR)	Achieved PASS, No. (%)
iHOT-33	42.9 (16.7)	41.2 (31.8-54.5)	31	54.2 (22.0)	53.6 (37.5-72.1)	103 (55)
HOS-ADL	72.9 (16.0)	76.5 (63.2-85.3)	38	77.1 (17.1)	80.9 (67.4-89.7)	89 (49)
Pain VAS	53.7 (23.1)	60 (35-72)	26	41.9 (25.4)	39 (20-66)	88 (49)

a HOS-ADL, Hip Outcome Score–Activities of Daily Living; iHOT-33, International Hip Outcome Tool–33; PASS, Patient Acceptable Symptom State; VAS, visual analog scale.

**Table 3:** Optimal Threshold for the PASS for Each Patient-Reported Outcome Measure^*a*^

	Score, Mean (SD)		ROC Curve Analysis			
	Did Not Achieve PASS (n = 102)	Achieved PASS (n = 98)	Optimal Final Score Threshold	Specificity, %	Sensitivity, %	AUC (95% CI)
iHOT-33	37.4 (13.8)	70.6 (15.0)	50	91	82	0.95 (0.92-0.97)
HOS-ADL	65.7 (16.5)	88.0 (8.5)	66	81	86	0.90 (0.86-0.94)
Pain VAS	58.9 (19.6)	25.8 (18.8)	36	67	85	0.86 (0.82-0.93)

a AUC, area under curve; iHOT-33, International Hip Outcome Tool–33; HOS-ADL, Hip Outcome Score–Activities of Daily Living; PASS, Patient Acceptable Symptom State; ROC, receiver operator curve; VAS, visual analog scale.

Our multivariable analysis demonstrated that age was associated with achieving PASS for iHOT-33 (odds ratio, 1.04; 95% CI, 1.01-1.08) (Table 4). Baseline symptom severity, as represented by baseline iHOT-33, was significantly associated with achieving PASS for all scores. Patient factors such as activity score, sex, and BMI were not significantly associated with achieving PASS for any of the PROMs, and neither were radiographic markers (impingement type, Tönnis classification grade, alpha angle, lateral center-edge angle) or symptom duration.

**Table 4:** Multivariable Logistic Regression Model Exploring the Association Between Patient Baseline Factors and Achieving PASS in iHOT-33, HOS-ADL, and Pain VAS^*a*^

	iHOT-33		HOS-ADL		Pain VAS	
	OR (95% CI)	*P* Value	OR (95% CI)	*P* Value	OR (95% CI)	*P* Value
Age	1.04 (1.01-1.08)	.017^*b*^	1.03 (0.99-1.06)	.14	1.01 (0.98-1.05)	.56
Sex: male vs female	0.72 (0.34-1.52)	.39	0.68 (0.32-1.42)	.31	1.43 (0.70-2.92)	.33
Body mass index	0.98 (0.91-1.05)	.56	0.99 (0.93-1.06)	.87	1.02 (0.96-1.09)	.57
Tegner score	1.19 (0.99-1.43)	.07	1.10 (0.91-1.32)	.32	1.15 (0.96-1.38)	.13
Impingement type vs mixed						
Cam	0.99 (0.46-2.14)	.55	0.95 (0.43-2.10)	.48	0.97 (0.46-2.06)	.70
Pincer	0.61 (0.21-1.75)	.33	0.51 (0.18-1.44)	.20	1.29 (0.47-3.54)	.59
Tonnis grade 1 vs 0	0.84 (0.39-1.84)	.67	0.79 (0.36-1.71)	.54	0.51 (0.24-1.10)	.08
Alpha angle	0.99 (0.95-1.05)	.65	0.99 (0.96-1.03)	.74	0.97 (0.93-1.01)	.10
LCEA	1.00 (0.96-1.05)	.87	1.00 (0.95-1.04)	.82	0.97 (0.93-1.02)	.26
Symptom duration, mo	1.00 (0.99-1.01)	.95	1.00 (0.99-1.01)	.98	1.00 (0.99-1.01)	.85
Baseline iHOT-33	1.07 (1.04-1.09)	<.001^*b*^	1.05 (1.03-1.08)	<.001^*b*^	1.05 (1.02-1.07)	<.001^*b*^

a HOS-ADL, Hip Outcome Score–Activities of Daily Living; iHOT-33, International Hip Outcome Tool–33; LCEA, lateral center-edge angle; OR, odds ratio; PASS, Patient Acceptable Symptom State; VAS, visual analog scale.

b *P* < .05.

## Discussion

We reported the 6-month PASS threshold score for common PROMs in a patient population with FAIS treated with a standardized physical therapy protocol. Our findings are important because they provide a benchmark for the PASS after nonoperative intervention. These values will better allow researchers and clinicians to assess the effectiveness of nonoperative interventions in allowing patients to achieve a state of wellness. Additionally, this may improve our ability to compare nonoperative interventions with surgical management outcomes and aid in the determination of appropriate interventions for patients with FAIS depending on their expectations and perceived level of wellness. Instead of assessing patient-related outcomes as mean (SD) within a single-group context, our reported benchmarks provide context at the individual level and allow for analysis in terms of proportions of patients who achieve the thresholds across treatment arms.

Previous studies have focused on the surgical population of FAIS and have identified increasing age, elevated BMI, female gender, arthritic changes/chondral injury, and radiographic deformities as factors associated with poorer outcomes overall without clear interpretation to whether they affect patient satisfaction.^28^ Maxwell et al^19^ did not identify any factors predicting achievement of PASS in their surgical cohort, including baseline iHOT-33 scores, age, sex, deformity, and chondral damage. On the contrary, Cvetanovich et al^7^ found that younger age, larger medial joint space, and higher baseline HOS-ADL scores predicted achieving PASS for HOS-ADL, and Kuhns et al^16^ reported that athletic patients were more likely to achieve PASS as compared with nonathletic populations after arthroscopic management of FAIS. In our study focusing on patients undergoing nonoperative treatment, baseline iHOT-33 scores were predictive of satisfactory outcomes. This may suggest that higher baseline function may predict greater patient-recognized success of nonoperative and surgical therapy. In contrast, patient factors including sex, BMI, and radiographic markers of disease severity or arthritic changes were not predictive of achieving PASS, suggesting that demographics and radiographic assessments should not prohibit a trial of nonoperative management, especially given the lower risks and costs as compared with surgical intervention.^10^

In our study, approximately a third of patients (31%, iHOT-33; 38%, HOS-ADL; 26%, pain VAS) had baseline scores already above the PASS threshold. This is much higher than the proportion previously reported in surgical cohorts (9%, iHOT-33; 0%, HOS-ADL; 0%, HOS–Sport Scale; 6%, modified Harris Hip Score).^18,19^ Despite these patients already meeting the PASS threshold at baseline evaluation, we can only postulate that they were not satisfied with their presenting symptom state given that they continued to seek clinical evaluation. This discrepancy may suggest that patients and surgeons are likely more willing to trial nonoperative treatment in patients who have a higher baseline function and a symptom state that approaches PASS thresholds.

Given the increased personal investment and risk experienced by patients undergoing surgery, we hypothesized that the PASS thresholds in our nonoperative cohort would be lower than those previously reported in cohorts undergoing hip arthroscopy surgery. Maxwell et al^19^ reported a PASS threshold of 58 for iHOT-33 at 2 years after surgery as compared with our determined threshold of 50. Ueland et al^35^ completed a systematic review of PASS thresholds in surgically managed FAIS cases including 4 studies that reported HOS-ADL scores. They determined a PASS threshold of 87 to 92 for HOS-ADL, again higher than our threshold of 66. There were no studies that examined the PASS threshold of pain VAS in the operatively treated FAIS cohorts. It appears that patients who are surgically managed must achieve higher PROM scores to be satisfied with treatment as compared with those treated nonoperatively.

From a clinical perspective, the PASS thresholds reported in this study provide a practical framework for interpreting PROM scores in patients undergoing nonoperative management of FAIS, while reinforcing the distinction between population-level research metrics and individual patient decision making. PASS represents the score above which the majority of patients consider their symptom state acceptable; however, it is not intended to define an absolute threshold for all individuals or to independently determine candidacy for surgical intervention. Some patients may have PROM scores at or above the PASS threshold yet remain dissatisfied owing to higher activity demands, expectations for symptom resolution, or quality-of-life goals. Accordingly, PASS should be used to contextualize PROM scores during shared decision-making rather than as a prescriptive treatment cutoff. Clinicians may use these thresholds to frame discussions regarding expected outcomes (eg, that most patients with scores above a given value report satisfactory symptoms), while continuing to individualize treatment recommendations based on patient goals, functional demands, symptom persistence, and response to previous nonoperative care. In this manner, PASS complements traditional PROM interpretation by shifting the focus from mean score changes to patient-perceived acceptability of symptoms, particularly in the nonoperative treatment setting where residual symptoms may still be compatible with a satisfactory clinical state.

### Limitations

Despite its strengths, our study has several limitations. First, the enrolled population was quite heterogeneous in terms of symptom duration and baseline activity level. This may limit the generalizability of our findings to particular groups (ie, high-level athletes), who may have different individualized thresholds for what is considered a state of wellness. Second, while patients were instructed to complete the exercise program 4 times per week, there was some variation in their regimen as well as in other activities beyond the prescribed program, from which they were not formally restricted. This again introduces an element of heterogeneity. Third, patients did not incur any financial costs from their participation in the physical therapy program, including the 2 in-person visits with a physical therapist. Depending on the health system in which patients exist, this may not be the experience for all those managed with nonoperative exercise-based care. Personal financial costs may affect patients’ view of investment in a treatment program and subsequently influence their perception of effectiveness and wellness. Little research has been done to evaluate the association between patients’ financial expectations and their perception of effectiveness and wellness. Fourth, we utilized a single anchor question to assess a general acceptable symptomatic state to establish PASS values in 3 PROMs. While this approach has been commonly utilized, utilizing separate anchor questions to address different physical constructs of a patient’s state may provide a more nuanced assessment of the patient’s condition. Finally, selection bias was a factor in our study, as patients not willing to participate in a formal nonoperative program were not included in our cohort.

## Conclusion

This study determined the PASS value for iHOT-33 (PASS = 50), HOS-ADL (PASS = 66), and pain VAS (PASS = 36) for patients with FAIS treated nonoperatively with an exercise-based, core-focused physical therapy program. The information in this study can be utilized by clinicians in counseling individual patients to anticipated outcomes and by investigators for future nonoperative-focused outcomes research.

## Supplemental Material

sj-pdf-1-ajs-10.1177_03635465261421191 – Supplemental material for The Patient Acceptable Symptom State for Commonly Used Patient-Reported Outcomes After Nonoperative Management of Hip Femoroacetabular Impingement Syndrome Supplemental material, sj-pdf-1-ajs-10.1177_03635465261421191 for The Patient Acceptable Symptom State for Commonly Used Patient-Reported Outcomes After Nonoperative Management of Hip Femoroacetabular Impingement Syndrome by Graeme Hoit, Daniel B. Whelan, Valerie Lemieux, Brent Bates, Tim Dwyer, John Theodoropoulos and Jaskarndip Chahal in The American Journal of Sports Medicine
