# Supplementary material for: The Patient Acceptable Symptom State for Commonly Used Patient-Reported Outcomes After Nonoperative Management of Hip Femoroacetabular Impingement Syndrome
Source: Am J Sports Med. 2026 Feb 24;54(5):1166–73. doi: 10.1177/03635465261421191 (PMC13031370; doi:10.1177/03635465261421191)
Supplement: sj-pdf-1-ajs-10.1177_03635465261421191 – Supplemental material for The Patient Acceptable Symptom State for Commonly Used Patient-Reported Outcomes After Nonoperative Management of Hip Femoroacetabular Impingement Syndrome [file sj-pdf-1-ajs-10.1177_03635465261421191.pdf]

The Patient Acceptable Symptomatic State for Commonly Used Patient-Reported Outcomes After Non-Operative Management of Hip Femoroacetabular Impingement Syndrome

Appendix

I. Table 1. Inclusion and Exclusion Criteria

Inclusion Criteria	Exclusion Criteria
- Age 16-55<br>- Clinical history in keeping with FAIS<br>- Radiographic signs of cam, pincer or mixed-type pathology<br>- Cam: alpha angle >55 degrees (Dunn and/or frog leg lateral x-ray) and/or pistol grip deformity (AP x-ray)<br>- Pincer: LCEA >40 degrees (AP x-ray), crossover sign (AP x-ray), ischial spine sign (AP x-ray), Tönnis angle <0 degrees (AP x-ray)<br>- Mixed: features of both cam and pincer morphology<br>- MRI evidence of labral tear	- Varsity or professional athlete<br>- Tönnis arthritis grade 2 or greater<br>- Lateral center edge angle <20 degrees (dysplasia)<br>- Previous hip pathology (Perthes, slipped capital femoral epiphysis, dislocation, avascular necrosis, fracture)<br>- Previous hip shape changing surgery<br>- Associated workplace insurance claim, motor vehicle collision claim, or other medicolegal claim<br>- Associated pain syndrome diagnoses (fibromyalgia, complex regional pain syndrome, inflammatory arthritis, daily opioid use, other chronic joint pain inhibiting ability to perform regular exercises [ie. chronic low back pain])
